# Five tips for conducting remote qualitative data collection in COVID times: theoretical and pragmatic considerations

**DOI:** 10.1590/1980-220X-REEUSP-2022-0277en

**Published:** 2023-05-08

**Authors:** Rhyquelle Rhibna Neris, Elizabeth Papathanassoglou, Ana Carolina Andrade Biaggi Leite, Cristina Garcia-Vivar, Francine DeMontigny, Lucila Castanheira Nascimento

**Affiliations:** 1 Escola de Enfermagem de Ribeirão Preto, Universidade de São Paulo, Ribeirão Preto, São Paulo, Brazil.; 2 University of Alberta, Faculty of Nursing, Edmonton, Canada.; 3 Neurosciences, Rehabilitation & Vision Strategic Clinic Network, Alberta Health Service, Edmonton, Canada.; 4 Universidad Pública de Navarra, Facultad de Ciencias de la Salud, Pamplona, Navarra, Spain.; 5 Université du Québec en Outaouais, Département des Sciences Infirmières, Gatineau, Canada.

**Keywords:** Data collection, Nursing Methodology Research, Qualitative research, Nursing research, COVID-19, Recolección de Datos;, Investigación Metodológica en Enfermería;, Investigación Cualitativa;, Investigación en Enfermería;, COVID-19, Coleta de Dados, Pesquisa Metodológica em Enfermagem, Pesquisa Qualitativa, Pesquisa em Enfermagem, COVID-19

## Abstract

**Objective:**

To provide five methodological and pragmatic tips for conducting remote qualitative data collection during the context of the COVID-19 pandemic.

**Method:**

The tips presented in this article are drawn from insights of our own experiences as researchers conducting remote qualitative research and from the evidence from the literature on qualitative methods. The relevant literature was identified through searches using relevant keywords in the following databases: CINAHL, PubMed, SCOPUS, and Web of Science. Searches were limited to articles in English and Portuguese, published from 2010 to 2021, to ensure a current understanding of the phenomenon.

**Results:**

Five tips are provided: 1) Pay attention to ethical issues; 2) Identify and select potential participants; 3) Choose the type of remote interview; 4) Be prepared to conduct the remote interview; and 5) Build rapport with the participant.

**Conclusion:**

Despite the challenges in conducting remote data collection, strengths are also acknowledged and our experience has shown that it is feasible to recruit and interview participants remotely. The discussions presented in this article will benefit, now and in the future, other research teams who may consider collecting data for their qualitative studies remotely.

## RESUMEN

**Objetivo:**

Proporcionar cinco consejos metodológicos y pragmáticos para el manejo de la recogida de datos cualitativos durante el contexto de la pandemia de COVID-19.

**Método:**

Los consejos presentados en este artículo son extraídos *de insights* de nuestras propias experiencias como investigadores que realizan investigaciones cualitativas remotas y de la evidencia de la literatura sobre métodos cualitativos. La literatura relevante fue identificada a través de búsquedas utilizando palabras clave relevantes en las siguientes bases de datos: CINAHL, PubMed, SCOPUS y Web of Science. Las búsquedas se limitaron a artículos en inglés y portugués publicados entre 2010 y 2021 para asegurar una comprensión actual del fenómeno.

**Resultados:**

Se ofrecen cinco consejos: 1) Preste atención a las cuestiones éticas; 2) Identifique y seleccione potenciales participantes; 3) Escoja el tipo de entrevista remota; 4) Esté preparado para coordinar la entrevista remota; y 5) Promueva el vínculo con su participante.

**Conclusiones:**

A pesar de los desafíos en el manejo de la recogida remota de datos, también se reconocen las fortalezas y nuestra experiencia ha demostrado que es viable reclutar y entrevistar participantes remotamente. Las discusiones presentadas en este artículo beneficiarán, ahora y en el futuro, a otros equipos de investigación que puedan considerar recopilar datos para sus estudios cualitativos de forma remota.

## RESUMO

**Objetivo:**

Fornecer cinco dicas metodológicas e pragmáticas para conduzir coleta remota de dados qualitativos durante o contexto da pandemia de COVID-19.

**Método:**

As dicas apresentadas neste artigo são extraídas de insights de nossas próprias experiências como pesquisadores conduzindo pesquisas qualitativas remotas e das evidências da literatura sobre métodos qualitativos. A literatura relevante foi identificada por meio de pesquisas usando palavras-chave relevantes nas seguintes bases de dados: CINAHL, PubMed, SCOPUS e Web of Science. As buscas foram limitadas a artigos em inglês e português publicados de 2010 a 2021 para garantir uma compreensão atual do fenômeno.

**Resultados:**

Cinco dicas são fornecidas: 1) Esteja atento às questões éticas; 2) Identifique e selecione potenciais participantes; 3) Escolha o tipo de entrevista remota; 4) Esteja preparado para conduzir a entrevista remota; e 5) Estabeleça previamente vínculo com o participante.

**Conclusão:**

Apesar dos desafios na condução da coleta remota de dados, os pontos fortes também são reconhecidos e nossa experiência tem mostrado que é viável recrutar e entrevistar participantes remotamente. As discussões apresentadas neste artigo beneficiarão, atualmente e no futuro, outras equipes de pesquisa que possam considerar a coleta de dados para seus estudos qualitativos remotamente.

## INTRODUCTION

The context of the COVID-19 pandemic led to a change in the world population’s behaviors, aiming at reducing the spread of the virus. Research procedures also had to be changed to suit this new world context^(1)^ and adapt to lockdown and mandatory social distancing measures^(2)^. Qualitative research involving data collection through face-to-face interactions has been significantly impacted^(3)^.

In contrast to all these challenges faced by researchers, the need for science to answer various research questions has not been suppressed during the pandemic. Qualitative research and the variety of research methodologies associated with it are adequate to answer these research questions and also necessary to address the unique experiences of individuals during the COVID-19 pandemic^(2)^. These methods allow us to understand what meaning there is in people’s health and illness processes^(4,5)^. Although qualitative researchers have long been utilizing remote methods to conduct qualitative research^(6)^, the COVID-19 pandemic has forced them to rapidly adapt their face-to-face methods and to explore remote methods^(7)^.

While social distancing triggered by the COVID-19 pandemic is decreasing dramatically in many countries, the lessons shared here can help researchers in future pandemics and emergencies. Additionally, conducting remote data collection can be challenging, particularly for a novice researcher. Challenges include paying attention to ethical issues of remote data collection, identifying and selecting potential participants, conducting remote interviews, preparing for the interview, and building good rapport with research participants. Given the lack of methodological studies guiding both novices and experienced qualitative researchers to conduct qualitative research, in this article we share key lessons learned based on our experience conducting qualitative data collection and on the extensive literature. This study aimed to provide five methodological and pragmatic tips for conducting remote qualitative data collection during the COVID-19 pandemic. The discussion presented in this article will benefit, now and in the future, other research teams who may consider collecting data for their qualitative studies remotely.

## METHODS

The tips presented in this article are drawn from insights of our own experiences as researchers conducting a research project called “Quality of life experience of adolescent, young adult survivors of childhood cancer and their families” during the ongoing pandemic as well as from the evidence from the literature on qualitative methods. The relevant literature to theoretical discussion was identified through searches with the following keywords: “Qualitative research”, “Qualitative study”, “Methods”, “Data collection”, “Methodology”, “COVID-19”, “Pandemic”. The Boolean operators AND and OR were used to structure the searches.

The databases searched were CINAHL (EBSCOhost), Pubmed/MEDLINE, SCOPUS (Elsevier), Web of Science Core Colletion (Clarivate Analytics). The searches were limited to articles in English and Portuguese that focused on data collection of qualitative methods and were published from 2010 to 2021. The rational for the selected languages of the studies was based on the researchers’ fluency. A manual search was performed to expand the scope of selected studies. This allowed identifying relevant articles that contributed to the results of this study, and which were not identified in the previous search carried out in the databases.

The study received institutional ethical approval (protocol number 012/2020) and all participants gave their consent when joining the research.

## RESULTS

### Tips for Conducting Remote Qualitative Data Collection

**Figure 1.**
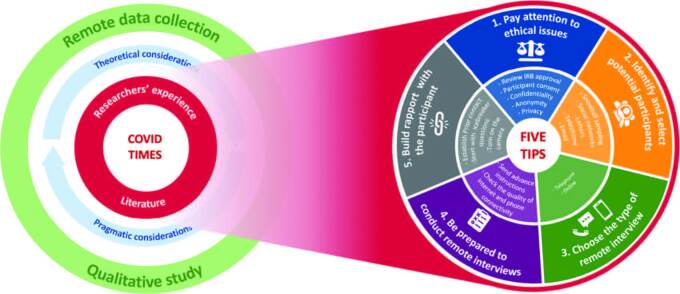
Tips to consider in the planning of remote qualitative data collection.

Five tips were identified, specifically: 1. Pay attention to ethical issues; 2. Identify and select potential participants; 3. Choose the type of remote interview; 4. Be prepared to conduct the remote interview; 5. Build rapport with the participant. Figure 1 illustrates these five tips that should be considered when conducting remote qualitative data collection.

### 1 – Pay Attention to Ethical Issues

Before starting data collection, it is critical to obtain institutional review board approval. Some ethical principles have to be kept in mind, regardless of the type of research, and cannot be neglected in remote qualitative data collection. They include: responsibilities for the dignity, privacy, and confidentiality^(8)^ of the participants as well as the assurance to “do no harm” to participants^(8,9)^.

The researcher has to prepare a consent form containing all the information about the study, ensuring the participant’s freedom to withdraw from the study at any point, and maintaining their anonymity. If the study has children and adolescents as a target population, the consent form must also be applied after officially obtaining the consent from the parents or legal guardians. Furthermore, the literature recommends that the researcher requests the participant’s written permission to audio record the interviews^(8)^.

In our practice during the COVID-19 pandemic conducting remote qualitative research, we prepared the consent form with Google forms and provided the access link to the potential participant. Using this option, consent is given after clicking on the option desired by the participant – “I accept to participate” or “I do not accept to participate in the research”. Ethics committees of some countries allow consent to be provided verbally, prior to the audio-taped interview.

The research risk for some participants is focused on the possibility of them remembering moments of vulnerability and suffering and being emotional, such as when narrating their cancer survival stories. In a remote interview, researchers should be extra vigilant in detecting any type of suffering from the participants^(7)^. In our experience, we have interrupted interviews to provide support to the participants through an empathetic and compassionate listening to their emotions. Additionally in a remote interview, the researcher can help the participant to identify sources of support to deal with the emotional moment after the interview. In the case of the pediatric studies, children and adolescents need to be free to choose whether they want their parents or guardians to be present during the interview. For some of them, the companions serve as a source of support, and for others, the presence of the parents hinders their interview.

Privacy of the information participants share with the researcher from their home is a significant ethical issue in remote data collection. Therefore, the researcher should ensure privacy by emphasizing that the interview should be conducted in a private and secluded room, whenever available, to reduce the chances of others overhearing the conversation with the researcher^(7)^. Respect for the participants’ anonymity is also an ethical issue that needs to be kept in mind for any research, and cannot be neglected in remote data collection^(10)^. Another critical ethical issue to be considered by the researcher is the risk of breach of confidentiality. Research files cannot be stored online, such as in online drives or cloud services. Some platforms automatically use these means to store interview recordings and the online consent form. In our practice, we download these materials to a local computer as soon as they are generated and immediately delete them from online drives or cloud services. It is recommended that the researcher only record the audio of the interview. However, most platforms simultaneously record MP4 video file format. The researcher has to use the audio for transcription and immediately delete the video, as this type of material hinders participants’ anonymity.

Another aspect often forgotten is that remote interviews give the researcher access to the participant’s home, which for certain persons might be perceived as a breach in their intimacy. Through this access, information related to the participant’s socio-economic living conditions is revealed. This intrusion in the participant’s environment must be respectful, and the information observed, kept confidential.

### 2 – Identify and Select Potential Participants

Remote recruitment carried out through social networks has increasingly presented itself as a potential source of research participants recruitment^(11)^. To recruit participants via social networking sites, the researcher has to be acquainted with virtual communities or support groups whose target audience is that relevant for the researcher and establish a partnership with the pages’ administrator for the dissemination of the research. The administrator may support the researcher in their role as key informant to spread the information about the research^(12)^. Another way to disseminate the information is through the creation, by the researcher, of an open social profile for the research, from which the researcher shares information about the research.

Recruitment through online media has the advantage of overcoming geographic limitations to include participants from different contexts^(13,14)^. However, it poses limits regarding accessibility to internet. Although this situation is changing, digital divide is a reality in many countries. This is of particular importance since access to virtual devices and the internet is greater for people with higher educational and economic levels. Researchers must be careful to mitigate this “digital divide” as best as possible, as it can exacerbate already existing health disparities^(7)^. Additionally, the researcher needs to consider the digital literacy of potential participants; for example, it can be a challenge to recruit elderly people through social networks, as part of this population still remains “offline”. Thus, when choosing recruitment through social networks, the researcher must assume this limitation.

Alternatively, participants can be sampled remotely through letters, telephone, and email. However, researchers have to consider the source of records they will refer to when extracting patient data and performing remote contact. Planning has to include prior ethical and institutional approval so that the researcher can have access to the data. The source of records may be public or private cancer treatment institutions; for example, the population-based registry of the Cancer Institute and cancer survivor support groups. When conducting recruitment, the researcher needs to provide participants with a detailed explanation of the purpose and procedures of the study, and also on how the researcher got access to their data.

Finally, the researcher can associate snowball sampling to recruitment. This sampling method has allowed us to recruit individuals for research based on the indication of a participant who had previously participated in the study. This sampling strategy has been described in the literature as useful for studying hard-to-reach groups^(15)^.

### 3 – Choose the Type of Remote Interview

Face-to-face interviews represent the foundation of qualitative research^(16)^. However, there are other approaches to interviewing, such as through telephone and online, which could be implemented by researchers during the COVID-19 pandemic to mitigate the challenges of physical distance in conducting qualitative research.

Online interviews can be conducted in different video platforms^(10)^. The literature points out that seeing and talking simultaneously decreases the awareness of the computer screen as a barrier to communication. Moreover, physical involvement neutralizes the participants’ anxiety^(17)^. A comparative study of face-to-face and video-call interviews using the same interviewer found modest differences between them, but the face-to-face one allowed for greater variation in statements in the data generated^(18)^. However, virtual methods may also present new privacy concerns as researchers may be intruding the participants’ personal space, especially if participants are in their own homes and do not use a virtual background or do not have access to headphones^(16)^.

Although video interviewing allows for observation of non-verbal responses to questions, the necessary technology may not be available or accessible in all circumstances and telephone interviewing may be more feasible^(16)^. This way is also viable for collecting data from participants who are geographically distant. Telephone interviews have the advantage that they can be conducted either using modern smartphones or devices with old technology^(17)^ and have low operational costs^(19)^. This form of interview allows participants to remain on “their own turf” and has a lower risk of loss of confidentiality^(19)^. Its challenge is the impossibility of visualizing the participant’s facial expressions and verifying whether the questions are causing the participant any suffering and the construction of rapport. However, at this moment of social distancing measures to minimize physical contact among individuals and restricted movement outside the home due to the COVID-19 pandemic, they are particularly suitable.

Conducting interviews remotely is a new scenario for many qualitative researchers; thus, due to inexperience, they can be excessively active during the interview. When conducting an interview to capture data for qualitative studies, interviewers should talk less and listen more to the participant. They should allow for silence to act as the catalyst that will drive the conversation forward^(20)^. During the remote interview, in which the researcher and participants are physically far away, the researcher has to show interest in the participant’s speech, but avoid interrupting until there are clear signs that the interviewee has finished their story^(21)^. The researcher may use non-verbal encouragement to show interest in the participant’s speech, e.g., smiling (if online interview), saying hmmm.

### 4 – Be Prepared to Conduct the Remote Interview

Based on our experience, conducting interviews online and by telephone is a time-consuming process. On the day of the interview, the researcher should check if everything is working fine for the time of the interview. It is useful for the researcher to recharge electronic devices’ battery before the interview, as battery shortage can interrupt a particpant’s important reflection. It is important to provide advance instructions to ensure better researcher-participant interaction during the interview. In remote interviews, in which the researcher and the participant are physically distant from each other, the researcher’s control over the interaction diminishes. To ensure successful moderation^(10,22)^, the set of instructions, including the request to minimize disturbing factors by turning off other potential apps and websites, social networks, silencing phones, among other things, to ensure a suitable environment for the interview. Being in a private setting can allow the participant to feel safe talking to the researcher about sensitive topics.

### Online Interview

To successfully conduct online interviews, the first thing the researcher has to check with the participants is availability of the necessary equipment, e.g., computer, smartphone or tablets with internet access, working camera, microphone, and headset. The use of a headset is useful to provide more privacy^(10)^. The researcher also has to check if the participant has good quality internet, but also needs to pay attention to the quality of their internet as well, to avoid glitches and unwanted drop-outs. In most cases, average quality is sufficient for participation with most video conferencing tools^(10)^. A poor connection during the interview can negatively impact the researcher-participant rapport^(13)^. In some cases, the research may provide participants with some mobile data to stimulate the participation^(12)^.

The researcher has to choose which video platform to be used together with the participant. It is always the researcher’s duty to certify which platform the participant feels comfortable using and schedule a test of the participant’s chosen video platform in advance. Examples of platforms that have been used successfully for qualitative data collection during the COVID-19 pandemic include: Zoom, Teams, Skype, WhatsApp, Messenger, Facetime, Webex, and Google Meet^(10,17)^. Although previous findings suggest the viability of Zoom as a tool for collection of qualitative data because of its relative ease of use, data management features, and safety options^(13)^, the basic free version of the platform limits each session to 40 minutes^(10)^ and the enhanced versions of the platform are paid. Therefore, researchers who have limited funding to develop their research may not be able to pay for these improved versions of the platform. In our experience, due to the familiarity with the participants, WhatsApp video calls and Google Meet video interviews were the preferred choice in our qualitative research.

All necessary guidance related to the participant’s chosen platform, such as: Does the participant have to create an account to access the platform? Is it necessary to download any app or perform any configuration to access the platform? If so, the researcher has to provide step-by-step instructions for using the platform or apps, starting with the installation of the apps on their smartphone, tablet, or laptop and how to use them.

The researcher should plan, in advance, how to record the audio of the interviews. Some platforms, e.g. Zoom, offer very easy recording functionality and split audio + video recordings into: audio; video; chat^(10)^. This makes it easier for the researcher to select the correct file to transcribe. If the participant chooses platforms that do not allow audio recording, for example WhatsApp, the researcher can put the phone next to the loudspeaker and record the interview externally with a tape recorder. It is important to certify that the devices are actually recording at the time of the interview and do back up recordings on the computer as soon as possible.

### Telephone Interview

In telephone interviews, a good telephone connectivity is required to ensure that the sound has good quality for transcription^(17)^. Therefore, the researcher has to check her/his and the participant’s telephone connectivity in advance. This may prove an issue for participants in areas with poor connectivity^(17)^.

The interviewer has to be ready and sensitive to listen to the interviewees’ actions, pauses, and omissions during the telephone interview. Telephone interviews may limit observation of non- verbal cues, so the researcher has to be aware of the participant’s voice intonation and pay attention to the ways they respond questions, for example, when the voice is loud or tearful^(12,17)^. However, telephones may allow respondents to feel relaxed and able to disclose sensitive information, and evidence is lacking on whether this format produces lower quality data^(19)^. It is also important to pay attention to the participant’s signs of tiredness in speech. The literature points out that telephone interviews tend to be shorter compared to face-to-face interviews^(19)^.

The way the interview will be recorded has to be planned. For example, the researcher can put the phone on the loudspeaker and externally record the interview with a tape recorder. However, it is important to do an audio test beforehand, the quality of the recording may not be high enough to allow for transcription afterwards. There are recording applications for mobile phones available (both for ios and Android), such as TapeaCall, Record my Call, and Call Recorder. These apps vary in their features, prices and availability depending on the researcher’s smartphone. Google Voice is a free service that allows recording interviews from any phone and send the MP3 files to the researcher’s email. The big disadvantage of Google Voice is that it only works with incoming calls, so the researcher has to ask the participant to call. The researcher has to make sure the devices are actually recording at the time of the interview and to make back up recordings as soon as possible on the computer.

### 5 – Build Rapport with the Participant

Building rapport and establishing good interactions in the qualitative interview situation is very important and is preferably done before the interview, but also in accordance with the literature during the interview itself^(20)^. Rapport is also crucial during the qualitative interview and allows the participant to provide a rich and detailed account of the experiences^(20)^. However, building rapport with the participant is a particular challenge of remote data collection. In our study, to mitigate this challenge, all data collection, from recruitment to interview, was conducted by the first author, whose strategy facilitated the construction of the rapport. In addition, the need to build rapport when the interview is conducted over the telephone is emphasized^(17)^.

Establishing prior contact, even remotely, can help overcome this challenge of having to build a researcher-participant rapport that is typical of the remote interview. Take the time to establish the rapport with the participant in advance, for example by explaining the project and data collection process to the participants. The key to building rapport is a sense of proximity^(20)^. At the time of the interview, the researcher can start the interviews with some chit-chat and ice-breaking questions to allow participants to feel comfortable and increase researcher-participant interaction. In our experience, it is common that at the beginning of the interview participants feel embarrassed and tend to provide short answers, as some participants may not feel comfortable with a video conversation.

For online interviews, it is important that the researcher and participant always turn on their video cameras throughout the interviews so that they can see, hear, and speak simultaneously. Our experience enables us to say that video allows us to build rapport with participants. The researcher was able to build a good rapport with the research participants, which can be confirmed with the production of in-depth and reflective reports about the researched experience. Good researcher-participant rapport was also evidenced by the request of some participants to perform a second interview. At the end of the interview, participants were asked for their opinions related to remote data collection, and they reported positive aspects related to remote data collection. The following two excerpts illustrate these statements: “I loved the research in the form of a video call, because even being far away, we are welcomed virtually, so through the video one can see the other, it allows a feeling of safety and comfort, more so in seeing who you are talking to. At the beginning (of the interview), I was a little embarrassed, but then I felt comfortable enough to talk. Even being far from each other, it gives that feeling of being side by side with the researcher. The only difference is that you don’t have a handshake and you don’t need to leave the house to go to the place to do the research” (adolescent, Burkitt’s lymphoma survivor, 16 years old). “I enjoyed participating. It was different to do research like this. Usually, we do it by phone or in person, I’ve never seen it by video. It was my first time and I really enjoyed it. It’s good that we managed to see each other even during the pandemic. Doing research in person is good, but you end up not saying everything right. I thought that by using my cell phone, I was more comfortable talking about what I wanted and I wasn’t afraid of the other person’s reaction” (adolescent, survivor of a pituitary germinoma, 17 years old).

## DISCUSSION

In this study, we provided five methodological and pragmatic tips for conducting remote qualitative data collection during the context of the COVID-19 pandemic. The tips presented in this article are drawn from insights of our own experiences as researchers conducting data collection with adolescents and young survivors of childhood cancer and from the literature. Methodological guidelines with tips to conduct remote qualitative data collection were limited in the literature.

As shown in several previous studies, remote interviews reduce the burden on participants, allowing them to participate in the research from the comfort of their own homes in a safe environment^(7,16,23)^. This is especially important considering the target population of our study, since cancer survivors may be at increased risk for severe COVID-19 due the numerous chronic comorbidities experienced after the intensive multimodality treatments^(24)^. Remote data collection also provided the researcher with a virus-safe socially-distanced environment.

Previous evidence on remote data collection have reported benefits such as: a) increased coverage of hard-to-reach populations such as those with mobility issues, rural populations, and those who live in communities with access to the Internet and technology but are geographically distant from the research site; b) opportunity for participants to participate from the comfort of their homes; c) reduced costs and logistical challenges; d) decreased peer pressure to provide socially desirable responses, and e) greater flexibility^(7,16)^. This is important considering that in some countries research centers are concentrated in more developed regions. However, it can promote a “digital divide”, including only participants who have immediate access to computers and the Internet^(7,25)^.

Special consideration should be given to ethical aspects when conducting remote data collection. Although remote data collection has the same ethical issues as face-to-face data collection^(10,25)^, studies have shown that online or telephone interviews present an additional risk of loss of confidentiality and privacy when compared to face-to-face interviews, as family members or others can hear what is being discussed^(7,17)^. Studies raise the ethical issues of involving research participants during a pandemic, which is a highly stressful and uncertain time, with life and normal routines disrupted. However, the interview can also be an opportunity for social support. Therefore, during remote data collection, researchers have to promote reflection and psychological well-being in the participants so that the interview becomes a therapeutic mechanism to deal with this moment of crisis^(17)^.

In our experience, one of the challenges of remote data collection was to keep participants motivated to conduct interviews after giving their consent. Although the researcher was careful to become familiar with the participant in advance, establishing prior contacts to build a good researcher-participant rapport, some participants did not respond to the researcher’s contact to perform the interviews. The literature has shown that even with extensive prior familiarization, each participant is unique and it is not possible to predict how the researcher-participant rapport will develop^(7,26)^. This dropout can also be due to the overload of online activities that the participants have experienced during the pandemic.

Online or telephone research without a video camera can prevent the researcher from making observations and capturing non-verbal cues^(7,12,17)^. In our experience, none of the research participants refused to turn on the camera and visual contact improved the quality of the interaction. A previous study reported different results, in which although participants were encouraged to turn on their video cameras, some refused to do so^(12)^. There was a fear of compromising their privacy if they turned the video camera on. However, the authors concluded that this situation did not compromise data quality as turning off the video camera allowed the participant to have greater openness to disclose confidential information^(12)^.

Finally, our experience has shown that remote recruitment and interviewing are promising alternatives for data collection during the current pandemic circumstances. Although remote data collection has been shown to be appropriate for interviewing cancer survivor adolescents and young adults about their quality of life experiences, this method may not be suitable for research involving other populations, such as participants with low digital literacy and/or an unstable clinical picture, such as participants dealing with chronic pain or terminality. Therefore, future research should apply this data collection method to other populations and age groups, to test its applicability in contexts different to those described in this study. The scientific community knows little about the impact of the COVID-19 pandemic on the process of conducting qualitative research, so more methodological research is needed to fill in the gaps and uncertainties that still remain.

## CONCLUSIONS

Despite the growing need to use remote methods to collect qualitative data brought about by the COVID-19 pandemic, methodological tips on these techniques in the literature are limited. When conducting remote data collection, the researcher needs to pay attention to ethical issues, identify and select potential participants, conduct remote interviews, prepare for the remote interview, and build rapport with the participant. Despite the challenges in conducting remote data collection, strengths are also acknowledged, and our experience has shown that it is feasible to recruit and interview participants remotely, and thus contribute to better qualitative knowledge of participants’ experience.

This study presents evidence to promote the use of tools for collecting qualitative data remotely, considering the new world context. Therefore, in the educational area, the tips can be applied to train, educate, and generate reflections and discussions among undergraduate and graduate students, as well as research group members. Finally, the practical and theoretical considerations presented in this study can help both experienced and novice researchers to conduct their qualitative research remotely now and in future pandemics and emergency situations with rigor and quality.
